# Dysregulation of FOXD2-AS1 promotes cell proliferation and migration and predicts poor prognosis in oral squamous cell carcinoma: a study based on TCGA data

**DOI:** 10.18632/aging.202268

**Published:** 2020-12-09

**Authors:** Zheqi Liu, Wenkai Zhou, Chengzhong Lin, Xiaoning Wang, Xu Zhang, Yu Zhang, Rong Yang, Wantao Chen, Wei Cao

**Affiliations:** 1 Department of Oral and Maxillofacial, Head and Neck Oncology, Shanghai Ninth People’s Hospital, Shanghai Jiao Tong University School of Medicine, Shanghai 200011 China; 2 Shanghai Key Laboratory of Stomatology, Shanghai Research Institute of Stomatology, National Clinical Research Center of Stomatology, Shanghai 200011, China; 3 Second Dental Clinic, Shanghai Ninth People’s Hospital, Shanghai Jiao Tong University School of Medicine, Shanghai 200011, China

**Keywords:** FOXD2-AS1, OSCC, prognostic model, proliferation, migration

## Abstract

FOXD2 adjacent opposite strand RNA 1 (FOXD2-AS1) plays an important role in the pathogenesis of some cancers. However, its functional role in oral squamous cell carcinoma (OSCC) remains largely unknown. In this study, we conducted expressional and functional analyses of FOXD2-AS1 using data from the Cancer Genome Atlas (TCGA) and *in vitro* OSCC assays. FOXD2-AS1 dysregulation was remarkably associated with radiation therapy, anatomic location, high histologic grade, and lymphovascular invasion (*P* < 0.05). A nomogram based on FOXD2-AS1 expression was constructed for use as a diagnostic indicator for OSCC patients, and multivariate cox regression analysis showed that FOXD2-AS1 expression was an independent prognostic factor for OSCC patients. KEGG, gene set enrichment analysis, and immune infiltration evaluations indicated that FOXD2-AS1 was involved in tumor progression via epithelial-to-mesenchymal transition and cell cycle regulation and was negatively associated with mast cell, DCs, iDCs, and B cells. FOXD2-AS1 silencing suppressed the proliferation and migration of Cal27 cells. Our findings showed that an aberrantly high FOXD2-AS1 expression predicts poor prognosis in OSCC; FOXD2-AS1 may act as an oncogenic protein by regulating cell proliferation and migration and may suppress adaptive immunity by modulating the number and function of antigen-presenting cells.

## INTRODUCTION

Oral squamous cell carcinoma (OSCC) is one of the most common malignant tumors in humans, accounting for 2% of all tumors. More than 350,000 new cases are diagnosed worldwide every year [1]. Despite the significant improvements in treatments, the 5-year survival rate still remains at only around 50%. With the great improvement in treatment modalities, including surgery, radiotherapy, chemotherapy and multidisciplinary comprehensive sequence therapy, the quality of life of OSCC patients has improved to a certain extent, but the 5-year survival rate still remains the same [2]. Distant metastasis has always been a major problem in the treatment of OSCC [3]. Although some progress has been made in the fields of tumor invasion and metastasis, the underlying molecular mechanisms still need to be explored. Therefore, a better understanding of the genetic and epigenetic molecular alterations in OSCC is the key to improve the prognosis of OSCC patients.

Long non-coding RNAs (lncRNAs) are transcripts of nucleotides measuring more than 200 bases in length, without protein-coding functions. Recent evidence shows that nearly 98% of genome transcripts in humans are non-coding RNAs (ncRNA) [4, 5]. Studies have demonstrated that lncRNAs play an important role in the progression of various types of tumors [6–9]. lncRNAs can act as molecular sponges, scaffolds, or guides for interaction with mRNAs, microRNAs, and proteins [10].

FOXD2-AS1 is a 2,527-bp lncRNA located on chromosome 1p33 and has been linked to several human cancers. The dysregulation of FOXD2-AS1 is involved in the modulation of various tumor-associated biological processes. FOXD2-AS1 facilitates non-small cell lung cancer progression via Wnt/β-catenin signaling [11], and its interaction with microRNA-185-5p contributes to colorectal cancer proliferation [12]. In addition, FOXD2-AS1 promotes gastric cancer progression by epigenetically silencing EphB3 via EZH2 and LSD1 [13]. Finally, FOXD2-AS1 has been found to facilitate cell migration by regulating the epithelial-to-mesenchymal transition (EMT) in colorectal cancer [14].

In this study, the prognostic value of FOXD2-AS1 was primarily evaluated in OSCC patients from the Cancer Genome Atlas (TCGA). Furthermore, a prognostic nomogram was constructed based on FOXD2-AS1 expression for prognostication. Moreover, we analyzed the potential mechanism of FOXD2-AS1 in OSCC using Gene Ontology (GO) and gene set enrichment analysis (GSEA).

## RESULTS

### High FOXD2-AS1 expression in OSCC

**Figure 1:**
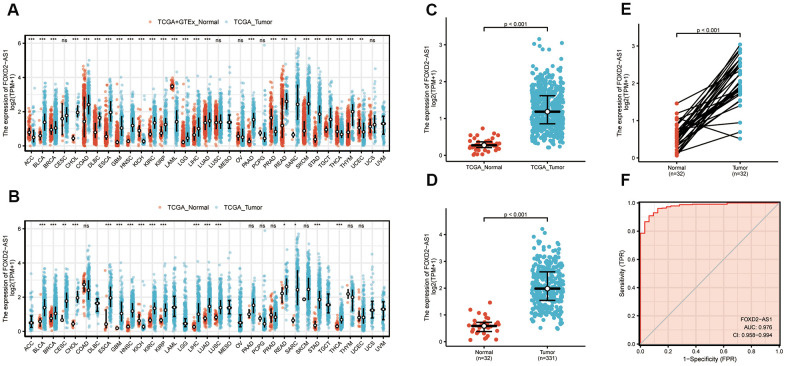
**Expression level of FOXD2-AS1 was upregulated in most cancer types including OSCC.** (**A**) FOXD2-AS1 expression in pan-cancer from normal TCGA samples of GTEx combined with samples of TCGA head and neck squamous cell carcinoma. (**B**) FOXD2-AS1 expression in pan-cancer of tumor tissues and paracancer tissues from TCGA. (**C**) FOXD2-AS1 expression of normal samples and tumor samples in TCGA head and neck squamous cell carcinoma. (**D**) FOXD2-AS1 expression of normal samples and tumor samples in OSCC patients. (**E**) FOXD2-AS1 expression of tumor tissues and paracancer tissues in OSCC patients. (**F**) ROC analysis of FOXD2-AS1 expression showing promising discrimination power between tumor and normal tissues.

To verify the FOXD2-AS1 expression in cancer, we first explored its expression using pan-cancer analysis, which included tumor tissues and paracancerous tissues. FOXD2-AS1 was upregulated in most cancer types (Figure 1A, 1B), including head and neck squamous cell carcinoma (HNSCC). The Wilcoxon rank-sum test was used to compare the expression of FOXD2-AS1 in normal, genotype-tissue expressed (GTEx) tumor samples with HNSCC samples from TCGA (Figure 1C). We excluded non-oral samples (Hypopharynx, Larynx, Oropharynx, Tonsil) in this analysis. A total of 331 OSCC patients with FOXD2-AS1 expression were obtained from TCGA. FOXD2-AS1 was significantly upregulated in tumor tissues relative to nontumor tissues (Figure 1D). We also compared the expression of FOXD2-AS1 in tumor tissues with their corresponding paracancerous samples and found that FOXD2-AS1 was highly expressed in the OSCC samples (Figure 1E). The receiver operating characteristic analysis revealed that FOXD2-AS1 can be used as a marker to distinguish tumor from non-tumor cells (Figure 1F).

### Association of FOXD2-AS1 expression with clinicopathological parameters

**Figure 2:**
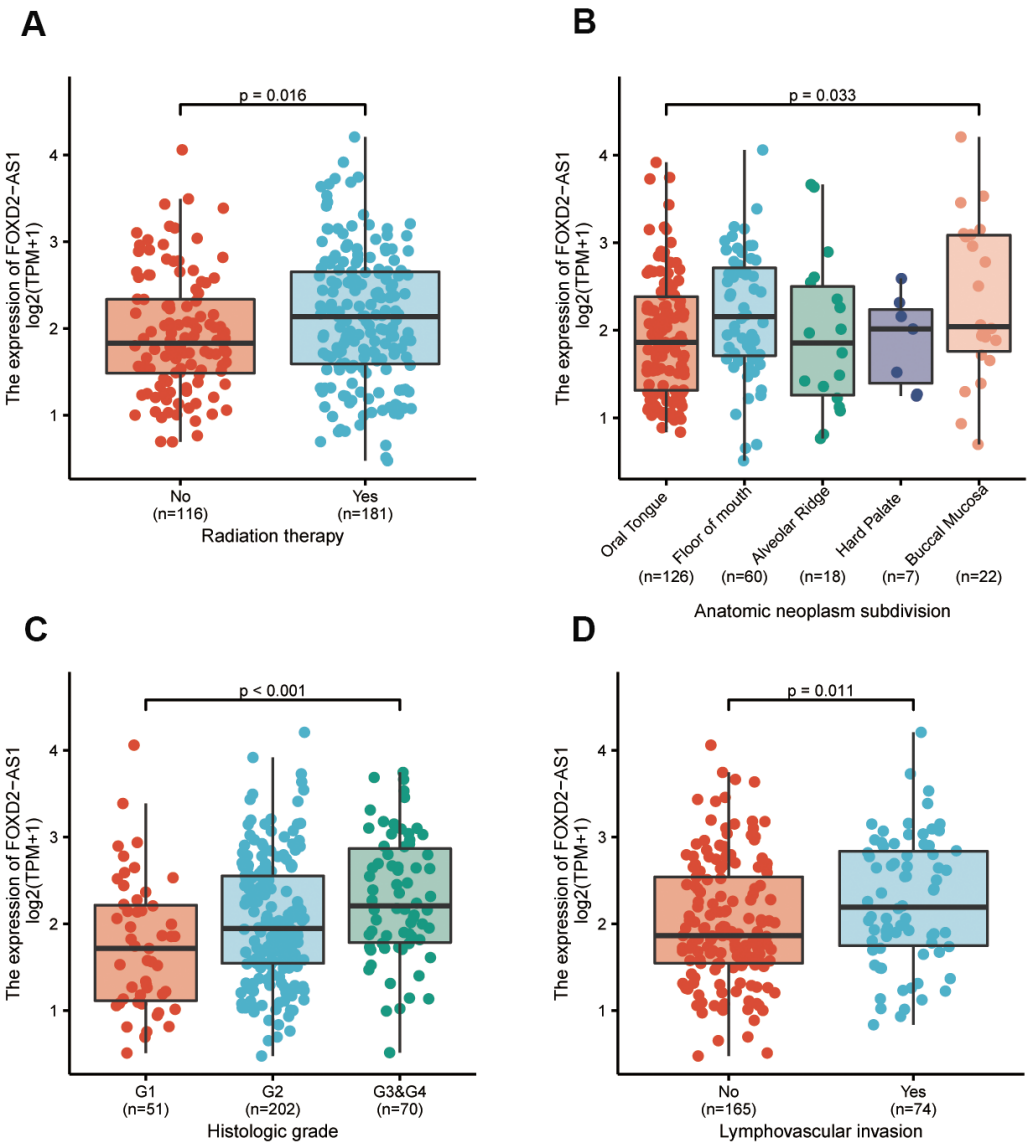
**The association between FOXD2-AS1 expression and clinicopathological parameters.** (**A**) The history of radiation therapy. (**B**) The anatomic localization of tumor. (**C**) The histologic grade of tumor. (**D**) The status of lymphovascular invasion.

To further elucidate the FOXD2-AS1 involvement in the development of OSCC, we analyzed the correlation of FOXD2-AS1 expression with clinicopathological parameters. In this study, the clinical information of 331 OSCC patients from TCGA were analyzed; among which, 228 male and 103 female patients with a median age of 62 years old (range: 52 to 71) were enrolled. Other clinicopathological features are shown in Table 1. The expression of FOXD2-AS1 in OSCC tissues was labeled as “low” or “high expression”, based on median values. High FOXD2-AS1 expression was strongly associated with radiation therapy, anatomic localization, high histologic grade, and lymphovascular invasion (*P* values were 0.016, 0.033, <0.001, and 0.011 respectively) (Figure 2A–2D). Other characteristics had no correlation with FOXD2-AS1 expression; detailed information is shown in Table 1. Logistic regression analysis of differential FOXD2-AS1 expressions is shown in Table 2.

**Table 1:** Characteristic of patients with OSCC based on TCGA.

**Characters**	**level**	**Low expression of FOXD2-AS1**	**High expression of FOXD2-AS1**	**p**	**Test**
n		166	165		
T stage (%)	T1	14(8.8%)	6(3.7%)	0.191	
	T2	55(34.6%)	50(30.9%)		
	T3	37(23.3%)	45(27.8%)		
	T4	53(33.3%)	61(37.7%)		
N stage (%)	N0	82(52.6%)	86(53.4%)	0.283	exact
	N1	34(21.8%)	24(14.9%)		
	N2	38(24.4%)	50(31.1%)		
	N3	2(1.3%)	1(0.6%)		
Clinical stage (%)	Stage I	9(5.7%)	2(1.2%)	0.105	exact
	Stage II	42(26.4%)	37(22.8%)		
	Stage III	34(21.4%)	34(21.0%)		
	Stage IV	74(46.5%)	89(54.9%)		
Gender (%)	Female	57(34.3%)	46(27.9%)	0.25	
	Male	109(65.7%)	119(72.1%)		
Age (%)	<=60	88(53.0%)	69(42.1%)	0.06	
	>60	78(47.0%)	95(57.9%)		
Histologic grade (%)	G1	32(19.6%)	19(11.9%)	0.019	exact
	G2	105(64.4%)	97(60.6%)		
	G3	26(16.0%)	42(26.2%)		
	G4	0(0.0%)	2(1.2%)		
Smoker (%)	No	49(29.7%)	38(23.8%)	0.278	
	Yes	116(70.3%)	122(76.2%)		
Alcohol (%)	No	53(32.7%)	53(32.9%)	1	
	Yes	109(67.3%)	108(67.1%)		
Age (median [IQR])	60.00[52.00,69.00]	63.00[55.00,71.00]	0.069	nonnorm

**Table 2:** FOXD2-AS1 expression associated with clinicopathological parameters (logistic regression).

**Characteristics**	**Odds ratio in FOXD2-AS1 expression**	**Odds ratio(OR)**	***P* value**
T stage (T3 and T4 vs. T1 and T2)	321	1.45(0.93-2.28)	0.105
N stage (N1 and N2 and N3 vs. N0)	317	0.97(0.62-1.50)	0.879
Clinical stage (Stage III and Stage IV vs. Stage I and Stage II)	321	1.49(0.91-2.44)	0.111
Histologic grade (G3 and G4 vs. G1 and G2)	323	2.00(1.17-3.48)	0.013
Perineural invasion (Yes vs. No)	251	0.84(0.51-1.38)	0.5
Lymphovascular invasion (Yes vs. No)	239	1.84(1.06-3.22)	0.032

### Prognostic value of FOXD2-AS1 in OSCC

**Figure 3:**
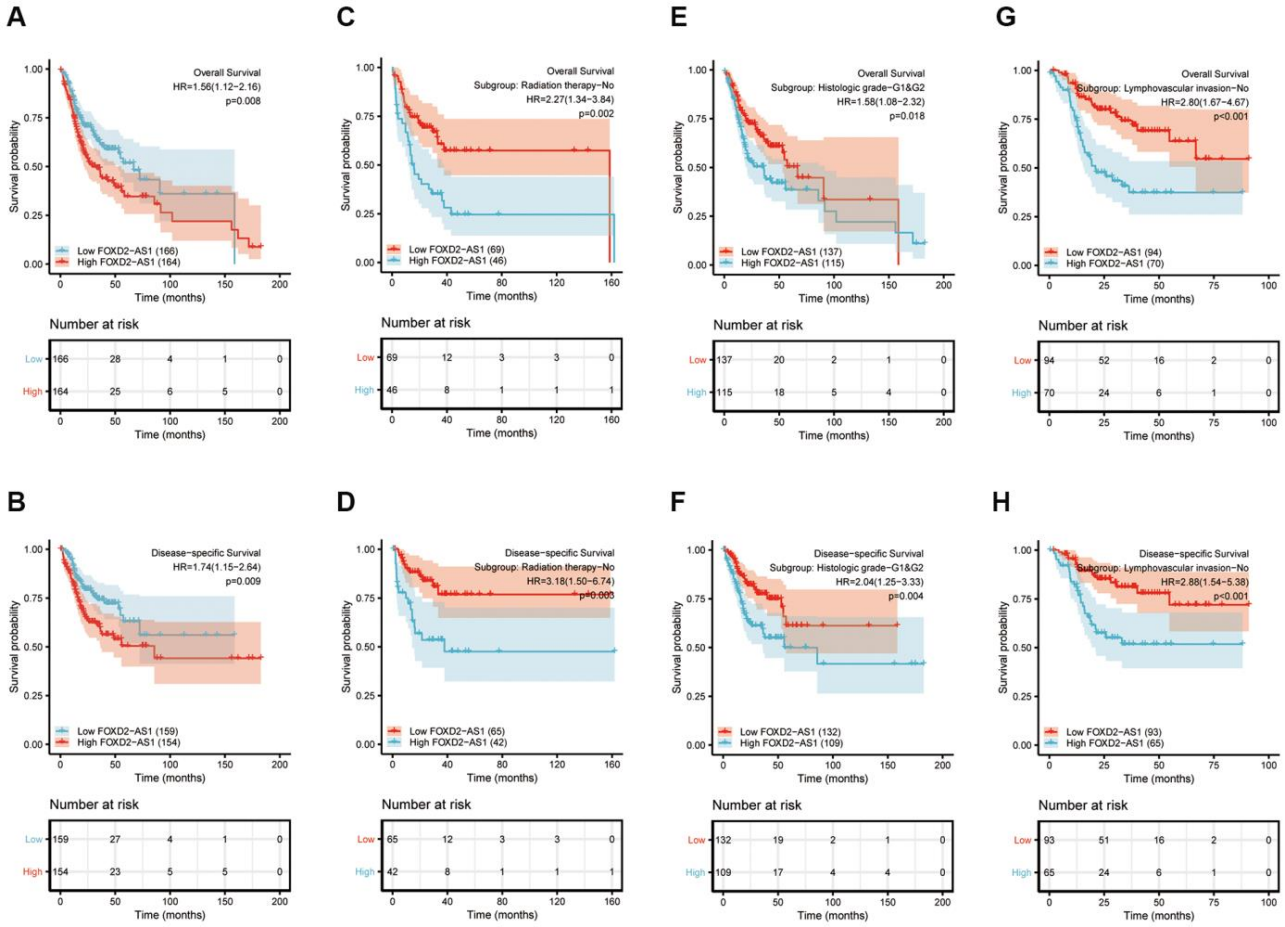
**Prognostic value of FOXD2-AS1 in OSCC.** (**A**, **B**) Kaplan-Meier curves of OS and DSS with different expression level of FOXD2-AS1. (**C**, **D**) Kaplan-Meier curves of OS and DSS with different expression level of FOXD2-AS1 in non-radiation therapy group. (**E**, **F**) Kaplan-Meier curves of OS and DSS with different expression level of FOXD2-AS1 in histologic grade I and grade II group. (**G**, **H**) Kaplan-Meier curves of OS and DSS with different expression level of FOXD2-AS1 in non-lymphovascular invasion group.

Kaplan-Meier curves of OS and disease-specific survival (DSS) were plotted to analyze the prognosis of OSCC patients using different expressions of FOXD2-AS1. Log-rank test of OS and DSS revealed that a high expression of FOXD2-AS1 was significantly associated to shorter survival (Figure 3A, 3B). We then performed a subgroup analysis to further explore the correlation between FOXD2-AS1 expression and patient prognosis with different characteristics. (Figure 3C–3H). The results showed that in non-radiated, histologic grades I and II, and non-lymphovascular invasive groups, a high FOXD2-AS1 expression predicted poor survival. Univariate Cox regression analysis revealed that perineural invasion, lymphovascular invasion, radiation therapy, and FOXD2-AS1 expression were prognostic factors for overall survival (OS) and DSS, while multivariate Cox regression showed that FOXD2-AS1 expression was an independent risk factor in OSCC patients (Tables 3, 4).

**Table 3:** Univariate and multivariate cox regression analysis of clinicopathological parameters and overall survival.

**Characteristics**	**Total(N)**	**HR(95% CI) univariate analysis**	**P value univariate analysis**	**HR(95% CI) multivariate analysis**	**P value multivariate analysis**
T stage (T3 and T4 vs. T1 and T2)	320	1.379(0.983-1.937)	0.063	1.749(1.018-3.004)	0.043
N stage (N1 and N2 and N3 vs. N0)	316	1.297(0.939-1.793)	0.115		
Clinical stage (Stage III and IV vs. Stage I and II)	320	1.269(0.880-1.829)	0.202		
Histologic grade (G3 and G4 vs. G1 and G2)	322	1.246(0.857-1.811)	0.249		
Age (>60 vs. <=60)	330	1.290(0.933-1.784)	0.123		
Smoker (Yes vs. No)	324	1.235(0.836-1.824)	0.288		
Alcohol history (Yes vs. No)	322	1.093(0.773-1.546)	0.615		
Perineural invasion (Yes vs. No)	250	1.924(1.282-2.886)	0.002	2.173(1.291-3.658)	0.003
Lymphovascular invasion (Yes vs. No)	238	1.671(1.119-2.496)	0.012	1.620(0.990-2.652)	0.055
Radiation therapy (Yes vs. No)	296	0.622(0.435-0.889)	0.009	0.452(0.265-0.770)	0.003
FOXD2-AS1 (High vs. Low)	330	1.556(1.121-2.160)	0.008	2.251(1.406-3.604)	<0.001

**Table 4:** Univariate and multivariate cox regression analysis of clinicopathological parameters and disease-specific survival.

**Characteristics**	**Total(N)**	**HR(95% CI) univariate analysis**	**P value univariate analysis**	**HR(95% CI) multivariate analysis**	**P value multivariate analysis**
T stage (T3 and T4 vs. T1 and T2)	303	1.788(1.129-2.833)	0.013	1.718(0.838-3.522)	0.139
N stage (N1 and N2 and N3 vs. N0)	299	1.570(1.037-2.376)	0.033	1.307(0.725-2.357)	0.373
Clinical stage (Stage III and IV vs. Stage I and II)	303	1.336(0.826-2.159)	0.238		
Histologic grade (G3 and G4 vs. G1 and G2)	308	1.327(0.837-2.104)	0.229		
Age (>60 vs. <=60)	313	1.170(0.776-1.763)	0.454		
Smoker (Yes vs. No)	308	1.196(0.740-1.933)	0.465		
Alcohol history (Yes vs. No)	306	1.482(0.922-2.384)	0.105		
Perineural invasion (Yes vs. No)	237	2.160(1.302-3.581)	0.003	2.304(1.187-4.471)	0.014
Lymphovascular invasion (Yes vs. No)	226	1.591(0.963-2.630)	0.07	0.957(0.540-1.694)	0.88
Radiation therapy (Yes vs. No)	286	0.837(0.525-1.335)	0.455		
FOXD2-AS1 (High vs. Low)	313	1.741(1.147-2.642)	0.009	2.513(1.430-4.416)	0.001

### Establishment of survival prognostic models for OSCC

**Figure 4:**
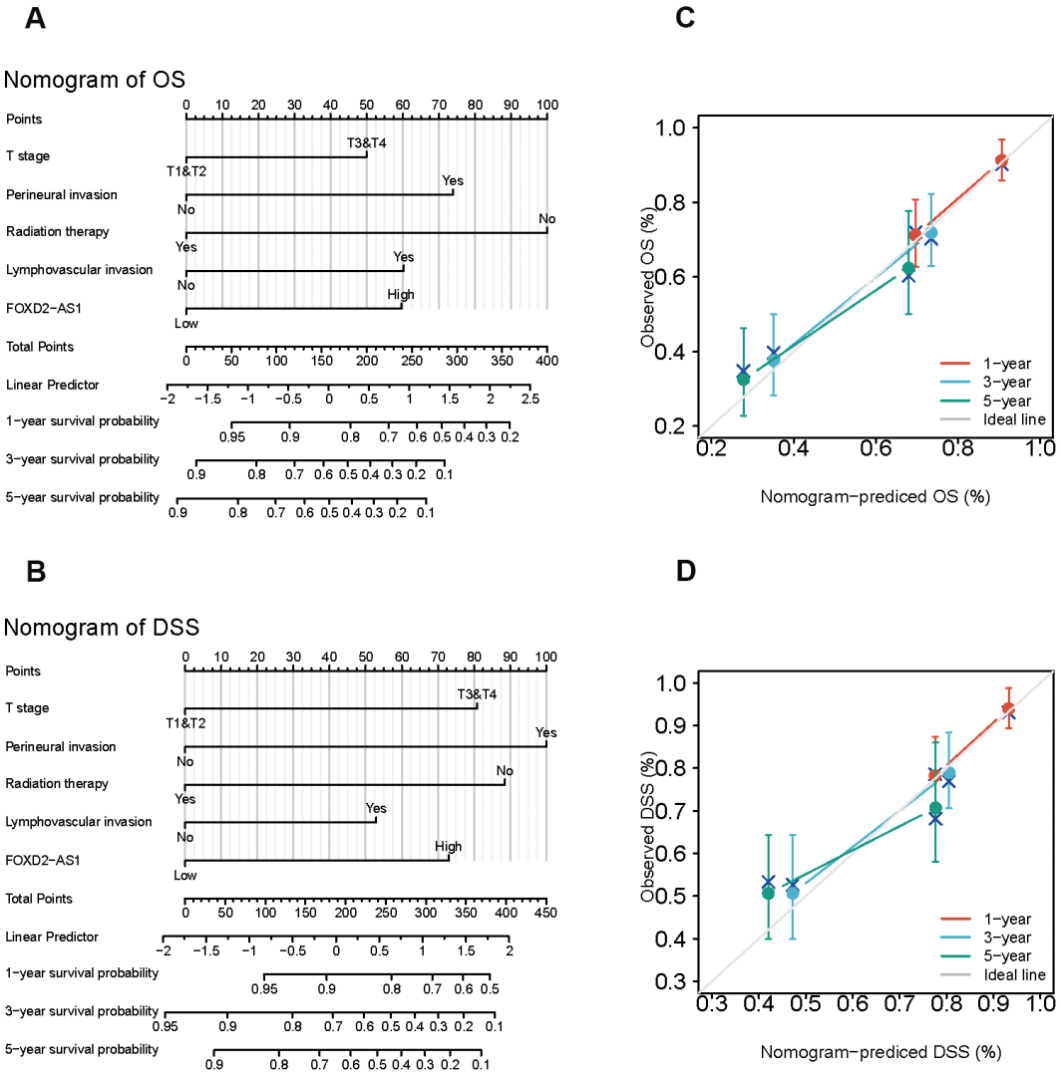
**Establishment and evaluation of survival prognostic models for OSCC.** (**A**, **B**) Nomogram for predicting the probability of 1-, 3- and 5-year OS and DSS for OSCC patients. (**C**, **D**) Calibration curve for evaluation the accuracy of nomogram of OS and DSS for OSCC patients.

Since the results mentioned above suggested that FOXD2-AS1 was an independent prognostic factor in OSCC, we tried to establish a prediction model of OS and DSS by fitting the expression of FOXD2-AS1 with the clinicopathological parameters. We constructed a nomogram to integrate FOXD2-AS1 and other prognostic factors, including the tumor (T) stage, perineural invasion, radiation therapy, and lymphovascular invasion (Figure 4A, 4B). A worse prognosis was represented by a higher point on the nomogram. The nomogram performance with FOXD2-AS was evaluated using a calibration curve, and the C-index of OS and DSS were 0.715 and 0.716, respectively (Figure 4C, 4D), suggesting that this nomogram might be a better model for predicting survival in OSCC patients than individual prognostic factors.

### The identification of differentially expressed genes (DEGs) in High FOXD2-AS1 expression and Low FOXD2-AS1 expression samples via functional cluster analysis

**Figure 5:**
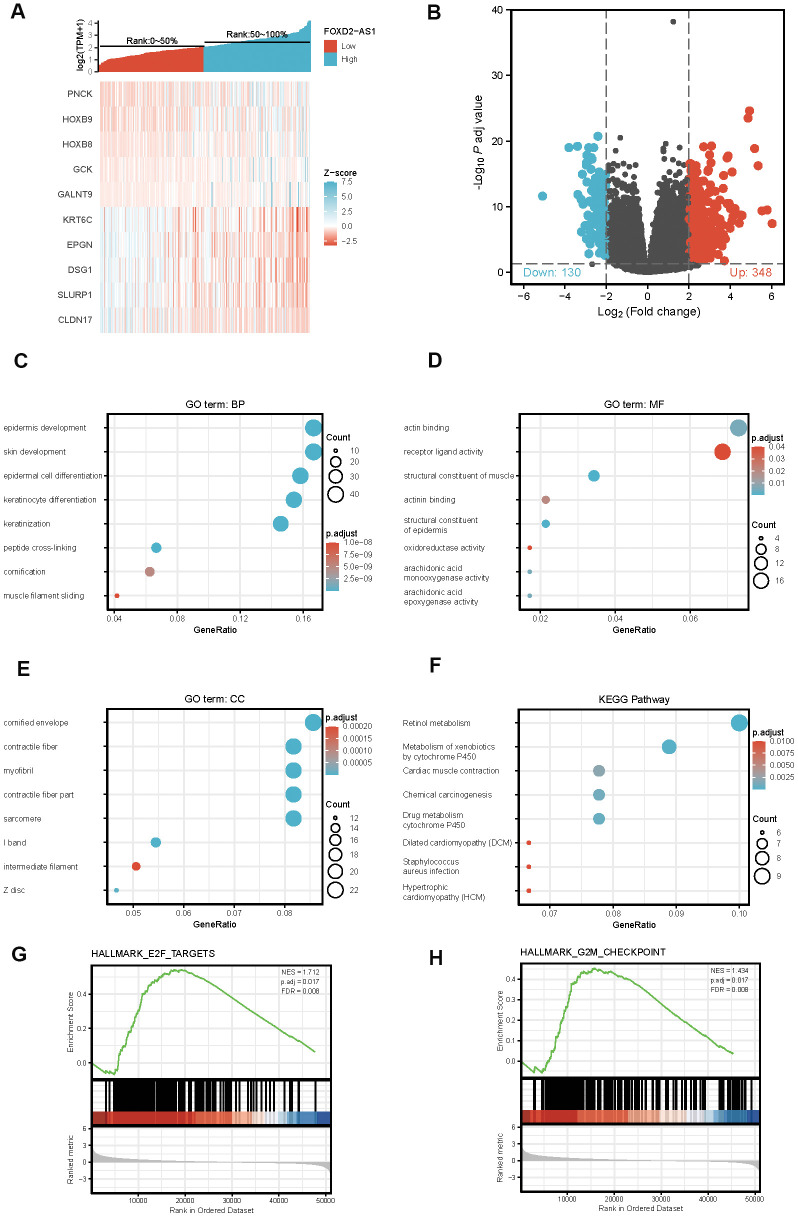
**Differentially expressed genes (DEGs) in High FOXD2-AS1 expression and Low FOXD2-AS1 expression samples and functional cluster analysis.** (**A**) Heat map of top 5 DEGs in high and low FOXD2-AS1 expression groups. (**B**) Volcano map of DEGs with |log2FoldChange >2| and adjusted P value<0.05. (**C**–**F**) Significantly enriched GO and KEGG annotations of FOXD2-AS1 related genes. (**G**, **H**) Enrichment of genes in E2F targets and G2/M checkpoint by GSEA.

To explore the potential mechanism of FOXD2-AS1 in causing tumor progression, we analyzed DEGs in high and low FOXD2-AS1 expression samples. A total of 478 DEGs were identified, with 348 upregulated genes and 130 downregulated genes. The expression of DEGs was demonstrated using a Heatmap and a Volcano Plot (Figure 5A, 5B). The functions of co-expression in OSCC patients were then predicted by GO and KEGG enrichment analyses. The top GO terms in the biological process (BP), molecular function (MF), and cellular component (CC) groups were keratinization, structural constituent of muscle, and cornified envelope, respectively. KEGG analysis revealed that the retinol metabolism was enriched (Figure 5C–5F). We also performed a GSEA to identify the key pathway related to FOXD2-AS1. The most significantly enriched pathways based on the NES were the E2F targets and G2/M checkpoint (Figure 5G, 5H).

### The correlation between FOXD2-AS1 expression and immune infiltration

**Figure 6:**
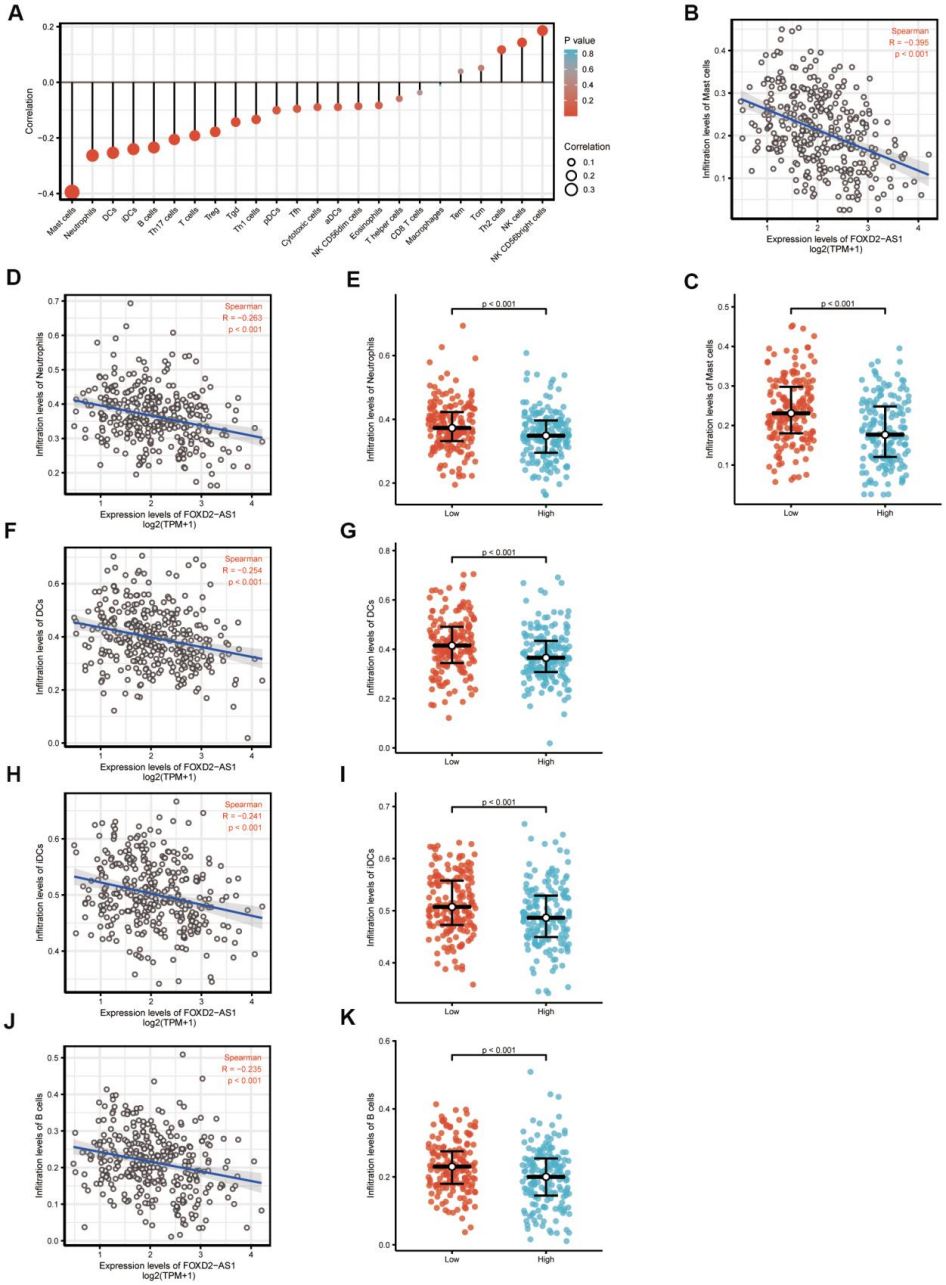
**The expression level of FOXD2-AS1 was associated with the immune infiltration in tumor environment.** (**A**) The plots showed the correlation between FOXD2-AS1 expression and immune cells subsets. (**B**) Spearman correlation between expression of FOXD2-AS1 and mast cells. (**C**)The plots of mast cells expression in low and high FOXD2-AS1 samples. (**D**–**K**) Spearman correlation and expression distribution of Neutrophils, DC cells, iDC cells and B cells in low and high FOXD2-AS1 samples.

We analyzed the correlation between FOXD2-AS1 expression and immune cell infiltration level (generated by ssGSEA) using Spearman correlation, as shown in Figure 6A. The results indicated that FOXD2-AS1 expression was strongly negatively correlated with mast cells. The Spearman R value was -0.395 with *P*<0.001. The Wilcoxon rank-sum test also indicated that the enrichment score of mast cells was higher in low FOXD2-AS1 expression group than in the high expression group (Figure 6B, 6C). Other immune cells with statistically significant enrichments were neutrophils, DCs, iDCs, and B cells (Figure 6D–6K).

### FOXD2-AS1 is upregulated in OSCC tissues and cell lines and promotes cell proliferation and migration *in vitro* and *in vivo*

**Figure 7:**
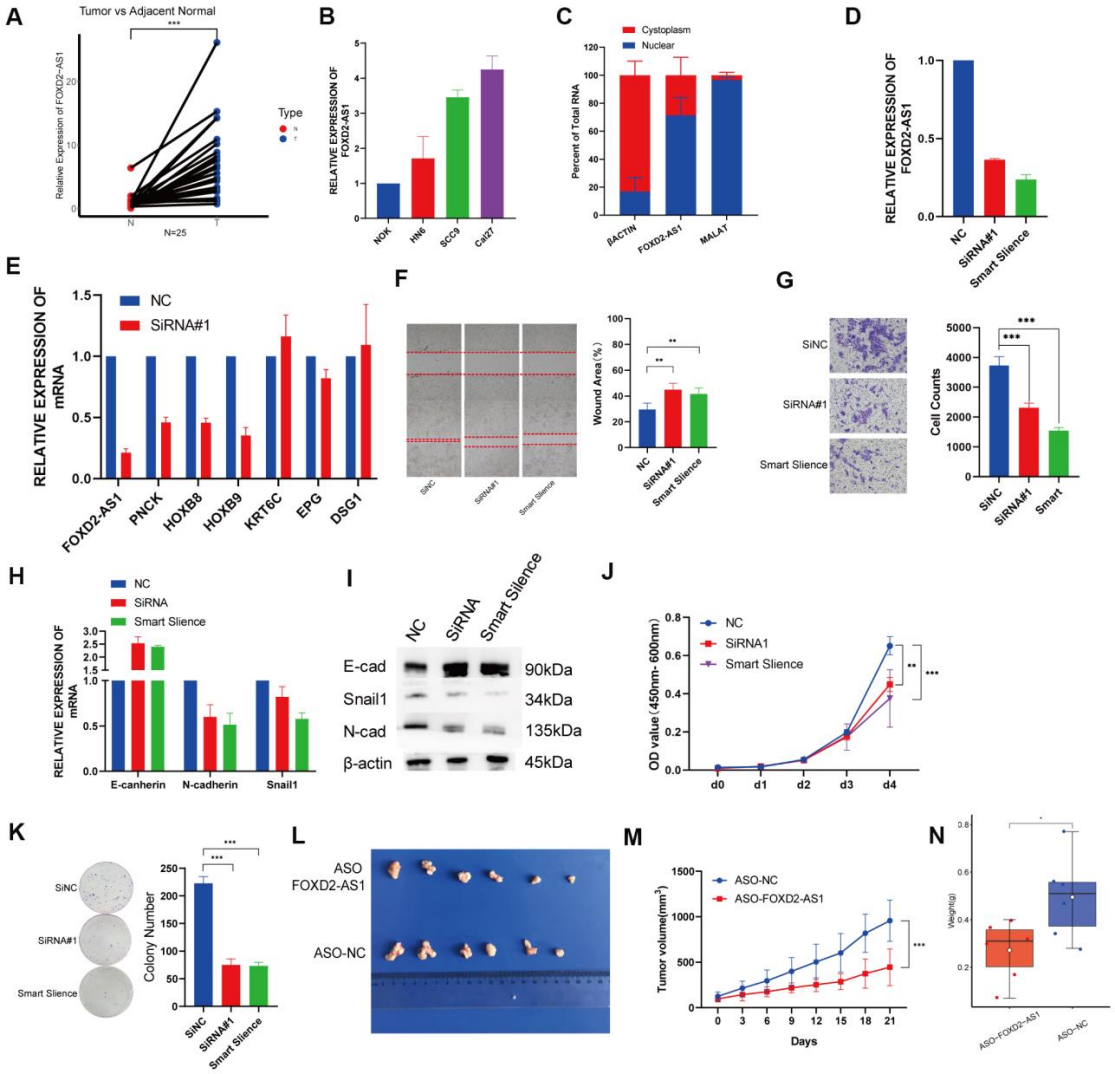
**Knockdown of FOXD2-AS1 decreased OSCC cell proliferation and migration *in vitro* and *in vivo*.** (**A**) The relative expression of FOXD2-AS1 in 25 paired tumor and adjacent normal tissues. The expression of FOXD2-AS1 was significantly up-regulated in Tumor tissues. (**B**) The relative expression of FOXD2-AS1 in 3 OSCC cell lines and Normal oral primary keratinocytes (NOK) cells. (**C**) Cell nucleus/cytoplasmic fractionation and qRT-PCR showed the subcellular localization of FOXD2-AS1 in Cal27 cells (MALAT1 and β actin were used as separation quality standards and endogenous controls). (**D**) The relative expression of FOXD2-AS1 after transfected with SiRNA and Smart Silence in Cal27 cell line. (**E**) The relative expression of PNCK, HOXB8, HOXB9, KRT6C, EPG and DSG1 after FOXD2-AS1 knockdown by SiRNA. (**F**) FOXD2-AS1 knockdown inhibited the migration capacity of Cal27 cells as detected by wound-healing assay after 48hs. (**G**) The cell migration abilities of Cal27 cells transfected with SiRNA and Smart silence were determined by transwell assays. (**H**) The relative mRNA expression of E-cadherin, N-cadherin and Snail in Cal27 cells transfected with SiRNA and Smart silence. (**I**) The protein expression of E-cadherin, N-cadherin and Snail in Cal27 cells transfected with SiRNA and Smart silence. FOXD2-AS1 knockdown inhibited the proliferation capacity of Cal27 cells as detected by CCK-8 assay (**J**) and colony formation assay (**K**). (**L**) Tumors were collected from different groups after 8 times ASO-FOXD2-AS1/ASO-NC injection. (**M**) Volume curve of tumors from different groups at the time of injection. (**N**) Weight of collected tumors from different groups. *p < 0.05, **p < 0.01, ***p < 0.001.

Twenty-five paired OSCC tissue samples and 3 OSCC cell lines were used to investigate the expression of FOXD2-AS1 via qPCR. FOXD2-AS1 expression was elevated in both tumor tissues and cell lines (Figure 7A, B). The subcellular localization was performed by qPCR and the results indicated that FOXD2-AS1 was localized predominantly in the nucleus (Figure 7C). Since the Cal27 cell lines had the highest expression of FOXD2-AS1, we performed a loss of function test in Cal27 using an siRNA and the Smart Silence Kit (Figure 7D). First, we validated the expression of the top 3 upregulated and downregulated genes filtered by TCGA. The results showed that the expression of PNCK, HOXB8, and HOXB9 was also decreased after the knockdown of FOXD2-AS1, while KRT6C and DSG1 were upregulated; this result was consistent with a previous TCGA analysis (Figure 7E). Next, we investigated the function of FOXD2-AS1 in the Cal27 cell line. Knockdown of FOXD2-AS1 remarkably impaired cell migration ability, as demonstrated in the wound healing and Transwell migration assays (Figure 7F, 7G). The mRNA and protein levels of N-cadherin and Snail1 were decreased, while the expression of E-cadherin was upregulated, as measured using qPCR and western blotting (Figure 7H, 7I). The silencing of FOXD2-AS1 also inhibited the proliferation of cancer cells according to the results of the CCK-8 and colony formation assays (Figure 7J, 7K). We also performed *in vivo* knockout experiments by injecting FOXD2-AS1 antisense oligonucleotides (ASOs) into nude mice. A xenograft tumor model was established via subcutaneous injection of Cal27 cells into nude mice. The injection of ASOs inhibited tumor growth (Figure 7L) and reduced tumor volume (*P* value < 0.001) (Figure 7M) and tumor weight (*P* value < 0.05) (Figure 7N). These results showed that FOXD2-AS1 was highly expressed in OSCC and can promote the proliferation and migration of cancer cells.

## DISCUSSION

Accumulating evidence has indicated that lncRNAs play an important role in regulating the pathophysiologic processes that occur in different cancers, including those of the oral squamous cell carcinoma [6, 9]. FOXD2-AS1 is a non-coding RNA, which is upregulated and involved in the regulation of disease progression in various cancers. FOXD2-AS1 can promote the progression of gastric cancer and non-small cell lung cancer by promoting cell entry into the cell cycle and reducing apoptosis [11, 13]. FOXD2-AS1 can also act as a sponge and abolish the endogenous suppressive effect of miRNAs on key targets in colorectal cancer, lung cancer, thyroid cancer, and gliomas [12, 15–17]. These studies indicated that FOXD2-AS1 is an oncogenic protein. However, the role of FOXD2-AS1 in OSCC has not been reported. In the present study, we aimed to elucidate the expression of FOXD2-AS1 in OSCC, its potential prognostic value, and probable regulatory mechanism.

In this study, the quantitative results indicated that FOXD2-AS1 had higher expression levels in most cancers including OSCC compared with normal tissues obtained from the TCGA database. The receiver operating characteristic (ROC) curve indicated that FOXD2-AS1 may be a diagnostic marker. Moreover, a high FOXD2-AS1 expression in OSCC was associated with poor differentiation grades, lymphovascular invasion, and poor prognosis. Univariate and multivariate cox regression showed that FOXD2-AS1 may be a promising prognostic biomarker for OSCC. Based on the results above, a prediction model for survival was established in OSCC patients, which might have an important clinical significance.

To further investigate the potential function of FOXD2-AS1 in OSCC, we analyzed the DEGs in low and high FOXD2-AS1 expression groups. The expression of 6 DEGs was validated via qPCR, and the expression changes of PNCK, HOXA9, HOXB8, KRT6C and DSG1 were consistent with the results of TCGA analysis. Among these upregulated DEGs, PNCK, HOXA9, and HOXB8 were all reported to be related to tumor progression in several cancers. PNCK was reported to promote proliferation in nasopharyngeal carcinoma cells [18]. The knockdown of HOXA9 inhibited cell proliferation, migration, invasion, and chemoresistance but promoted apoptosis in HNSCC cells. HOXA9 knockdown was also shown to regulate EMT-related markers by targeting YAP1/β-catenin [19]. HOXB8 was also reported to be an oncogene in gastric cancer and colorectal cancer by promoting tumor proliferation and EMT [20, 21]. On the other hand, among the downregulated DEGs, KRT6C, EPGN, and DSG1 were involved in the keratinization of the epithelium [22–26]. KRT6C was found to be associated with the prognosis and modulation of EMT in lung adenocarcinoma [23]. EPGN is a ligand of epidermal growth factor receptor (EGFR), which was known to be related with EMT and tumor metastasis. A lower expression of NSG1 is associated with poor survival in HNSCC [27]. One study showed that DSG1 can regulate invadopodia formation by suppressing EGFR/Erk signaling, the pathway that promotes keratinocyte differentiation, by interacting with the ErbB2-binding protein Erbin (ErbB2 Interacting Protein) [28]. These DEGs were more or less associated with tumor proliferation and EMT, suggesting that FOXD2-AS1 may promote OSCC by regulating cell proliferation and EMT. The results of the KEGG pathway analysis showed enrichment in the retinol metabolism. Retinoids, metabolites, and synthetic derivatives of vitamin A (retinol) have been shown to inhibit carcinogenesis in various epithelial tissues. A study from Guo et al. indicated that the reduced ability to esterify retinol in epithelial tumor cells might result in an inappropriate cell growth and the loss of normal differentiation [29]. Studies have shown that the all-trans retinoic acid (ATAR) can reverse EMT in cancers [30, 31], which is an important mechanism that facilitates migration and invasion in OSCC. The enrichment results, as well as previous literature, suggest that FOXD2-AS1 may promote OSCC via EMT. In our *in vitro* study, FOXD2-AS1 knockout impaired the migration ability of OSCC cell lines and altered the relative expression of E-cadherin, Vimentin, and Snail. The protein levels of these EMT biomarkers will be validated in a future study.

We also performed a GSEA analysis to search for the potential pathways affected by FOXD2-AS1. The results indicated that FOXD2-AS1 may affect OSCC progression through E2F targets and G2/M checkpoint, which are related to the cell cycle and cell proliferation. E2Fs have emerged as major transcriptional regulators of cell cycle-dependent gene expression [32]. E2Fs play an important role in the cell cycle, especially in the G1 to S phases, by controlling genes that encode DNA replication and regulate the cell cycle [33–36]. Su et al. have reported that FOXD2-AS1 can promote bladder cancer progression and recurrence through a positive feedback loop involving Akt and E2F1 [37]. In our study, we found that cell proliferation decreased after silencing FOXD2-AS1. Future cell cycle and cell apoptosis studies will be done to verify the mechanisms by which FOXD2-AS1 regulates these pathways.

Tumor-infiltrating immune cells have been shown to provide prognostic values in several human malignancies [38–40]. We analyzed the correlation between FOXD2-AS1 and these immune cells. The results showed that FOXD2-AS1 was negatively associated with mast cells, DCs, iDCs, and B cells. These immune cells can act as antigen-presenting cells (APCs) that ingest, process, and present extracellular antigens; activate CD4+T cells; and induce an immune response. DC numbers have been reported to be reduced in oral submucous fibrosis and OSCC [40]. A high FOXD2-AS1 expression level was related to the reduced APCs in our results, suggesting that FOXD2-AS1 may suppress the adaptive immunity and killer T-cell function by inhibiting the function and proliferation of APCs. However, the detailed mechanism behind this needs to be further investigated.

Overall, our findings suggest that high FOXD2-AS1 expression was an adverse prognostic factor of OSCC and shed light on the potential mechanism of FOXD2-AS1 in OSCC. Nevertheless, this study has some limitations. First, the data was accessed from a public database, and thus, the quality of data cannot be determined. Second, the mechanism of FOXD2-AS1 was predicted using bioinformatic approaches. Although we have validated the expression of FOXD2-AS1 *in vivo* and *in vitro* and verified a part of the function of FOXD2-AS1, further details of the molecular mechanism by which it affects the DEGs should be investigated using *in vitro* and *in vivo* experiments.

In summary, our results showed that patients with high FOXD2-AS1 levels had worse overall survival than those with low FOXD2-AS1 levels in the cohort. Through enrichment analysis, we determined that FOXD2-AS1 may act as an oncogenic factor by regulating the EMT of tumor cells and by suppressing adaptive immunity through the inhibition of the function and proliferation of APCs. Furthermore, the silencing of FOXD2-AS1 suppressed the proliferation and migration of OSCC cells and remarkably reduced the expression of Vimentin *in vitro*. Our findings revealed the roles of FOXD2-AS1 in OSCC and suggested that FOXD2-AS1 may be a potential biomarker for OSCC diagnosis and prognostication.

## MATERIALS AND METHODS

### RNA-sequencing data from TCGA

Gene expression data and corresponding clinical information of OSCC patients (331 cases, Workflow type: HTseq-FPKM) were downloaded from the HNSCC projects of TCGA (https://genome-cancer.ucsc.edu/). Patients diagnosed with OSCC with complete follow-up information were included. Next, the level 3 HTseq-FPKM data were transformed into transcripts per million reads (TPM) for further analyses. Unavailable and unknown clinical features were regarded as missing values. This study met the publication guidelines stated by TCGA. All data used in this study were obtained from TCGA, thus, ethics approval and informed consent were not required.

### DEG analysis

The samples were divided into high- and low-expression groups according to the level of FOXD2-AS1 expression (median as cut-off value). The expression data (TPM) of the high and low FOXD2-AS1 expression groups were then compared to identify DEGs using the limma package in R [41]. log_2_(Fold Change) >2 and adjusted *P* value <0.05 were set as threshold values for DEGs.

### Metascape analysis

Metascape, a free, well-maintained, and user-friendly gene list analysis tool for gene annotation and analysis [42], was used to conduct pathway and process enrichment analyses of DEGs. The conditions for differences to be deemed statistically significant included a *P* value <0.01, minimum count of 3, and an enrichment factor >1.5.

### Gene set enrichment analysis

GSEA was used to elucidate the significant function and pathway difference between high- and low- FOXD2-AS1 groups. Gene set permutations were performed 1000 times for each analysis. The expression level of FOXD2-AS1 was used as a phenotype label. Adj. *P* value< 0.05 and FDR< 0.25 were identified as significantly related genes. Statistical analysis and graphical plotting were conducted using the R package clusterProfiler [43].

### Analysis of immune infiltration and its correlation with FOXD2-AS1 expression

The immune infiltration analysis of OSCC was conducted via the single sample GSEA (ssGSEA) method using the GSVA package (http://www.bioconductor.org/packages/release/bioc/html/GSVA.html) in R. Based on the signature genes of 24 types of immunocytes in literature [44], the relative enrichment score of every immunocyte was quantified from gene expression profiles of each tumor sample. Wilcoxon rank-sum test and Spearman correlation were used to evaluate the association between FOXD2-AS1 expression and the 24 types of immune cells.

### Tissue samples and cell culture

Paired, fresh OSCC tissues and their corresponding adjacent normal tissues were collected from the Ninth People’s Hospital, Shanghai Jiao Tong University School of Medicine. Written informed consent was obtained from each patient. The patients were well-informed and have consented to this study, and the process was approved by Ethics Committee of the Ninth People’s Hospital, Shanghai Jiao Tong University School of Medicine.

Cal27, HN6, and SCC9 cells and normal oral primary keratinocytes (NOK) were used in this study. Cal27, SCC9 and HN6 were purchased from the Type Culture Collection of Chinese Academy of Sciences (Shanghai, China). NOK were cultured from gingival tissues after tooth extraction from healthy patients. These cell lines were maintained in DMEM (Basalmedia, Shanghai) supplemented with 10% fetal bovine serum, 1% glutamine, and 1% penicillin/streptomycin. Cells were cultured in a standard humidified atmosphere with 5% CO_2_ at 37° C.

### RNA extraction and qRT-PCR

Total RNA was extracted using TRIzol (Invitrogen, CA, United States). An equal amount of RNA was reverse-transcribed using the HiScript II Q RT Supermix and was quantified by qPCR using SYBR Green (Bimake). The primer sequences are shown in Supplementary Table 1.

### Western blotting

Cells transfected with SiRNA or the Smart Silence Kit for 48 h in 6-well plates were rinsed with PBS. Then, 150 μL of 1× SDS Protein Loading Buffer containing protease and phosphatase inhibitors were added to each well. Cell lysates were denatured at 98° C for 10 min and were subjected to 10% SDS-PAGE. The protein bands were then transferred to PVDF membranes, which were then blocked with 5% skimmed milk for 1 h and incubated with antibodies at 4° C overnight. The antibodies used in western blotting included those targeting β-actin (ABclonal AC026), E-cadherin (ABclonal A11492), N-cadherin (ABclonal A3045), and Snail (ABclonal A11794). HRP-conjugated secondary antibodies were used for detection.

### Isolation of nuclear and cytoplasmic RNA

Nuclear and cytoplasmic RNA was isolated using the PARIS™ kit (Thermo Fisher Scientific) according to the manufacturer’s instructions. The isolated RNAs were reverse transcribed and amplified via qPCR as described above. MALAT1 were used as an endogenous nuclear control, while β-actin were used as an endogenous cytoplasmic control.

### RNA interference

SiRNA against FOXD2-AS1 was designed and synthesized by Genomeditech Shanghai. The Smart Silence Kit was designed and synthesized by Guangzhou RiboBio Co., Ltd. (Guangzhou, China). These were transfected into the cells with Lipofectamine 3000 reagent (Invitrogen) according to the manufacturer’s instructions. The sequences are provided in Supplementary Table 2.

### Cell counting kit-8 (CCK-8) analysis

Cells transfected for 24 h with Smart Silencer/SiRNA were seeded into 96- well plates at a density of 1000 cells/well in triplicate. 10 μL of CCK-8 reagent (Dojindo, Kumamoto, Japan) was added to 100 μL of culture medium. The cells were subsequently incubated for 2 h at 37° C, and the optical density was measured at 450 nm and 600 nm using a microplate reader (Multiskan™ Sky Spectrophotometer, Thermo Scientific, USA).

### Colony formation assay

Cells transfected for 24 h with Smart Silencer/SiRNA were seeded into 6-well plates at a density of 1000 cells per well and incubated for 10–14 days to form cell colonies. The colonies were fixed with 4% paraformaldehyde (Sangon Biotech, Shanghai, China) for 15 min and stained with Giemsa stain (Solarbio, Beijing, China) for 30 min; those with more than 50 cells were counted under a dissecting microscope.

### Wound healing assay

The Cal27 cells were seeded into 6-well plates and transfected when cultures reached 50% confluence. The cell layers were then scratched using a 10-μL plastic pipette tip to produce wounds, which were photographed at 0 and 48 h under an inverted phase-contrast microscope. Three random fields were marked and measured. All assays were carried out in triplicate.

### Transwell migration assay

Cell migration assays were performed using 24-well Transwell chambers with 8-μm porosity polycarbonate filters and Transwell insert chambers (Corning, USA). A total of 200 μL of cell suspension in serum-free medium was added into each upper chamber, while 500 μL of DMEM supplemented with 10% FBS was added to the lower chambers as a chemoattractant. After incubating for 24 to 36 h, the migrated cells were fixed with 4% paraformaldehyde (Sangon Biotech) for 15 min and stained with Giemsa stain (Solarbio) for 30 min. After the cells on the upper surface of the filter were removed, at least 3 randomly selected microscopic fields of the fixed cells per filter were imaged using an inverted phase-contrast microscope. The cells were counted and cell counts were averaged.

### *In vivo* assays

SPF nude mice (5 weeks) were purchased from the Shanghai Laboratory Animal Center (Shanghai, China) and housed under SPF conditions at the animal care facility of the Experimental Animal Center of Shanghai University of Traditional Chinese Medicine. A total of 2×106 Cal27 cells in 200 μL serum-free DMEM were subcutaneously injected into the flanks of the nude mice. The initial tumor xenograft volumes were similar. When the tumor size reached 100 mm^3^, the mice were randomized into 2 treatment groups: (a) ASO-NC group (5 nM/3 days); (b) ASO-FOXD2-AS1 group (5 nM/3 days). The ASO used was chemically modified by Ribobio. Its sequence was the first one of the Smart Silence Kit. Tumor size and animal weight were monitored at the time of ASO injection, and tumor volume was calculated. Three days after the 7^th^ injection, the mice were sacrificed and tumor tissues were collected and weighed.

### Statistical analysis

Statistical analysis was performed using R(3.6.1). A comparison between the FOXD2-AS1 expression in tumor and normal tissues was performed using Wilcoxon rank-sum tests. The samples were divided into high- and low-expression groups (median FOXD2-AS1 expression level as cut-off value). Next, the ROC curve was made to test the performance of FOXD2-AS1 as a diagnostic marker using the survivalROC R package. The relationships between clinicopathologic features and FOXD2-AS1 expression were analyzed using the Wilcoxon rank-sum test and logistic regression. Clinicopathologic characteristics associated with OS and DSS were analyzed with Cox regression and the Kaplan-Meier method. The multivariate Cox analysis was used to identify the influence of FOXD2-AS1 expression on survival, along with other clinical features. A nomogram was constructed based on the results of the multivariate analysis using the rms package in R. A risk score for each patient was calculated as the sum of the scores for each parameter, which were obtained by multiplying the value or level of each parameter and its coefficient. According to the median risk scores, patients were divided into low- and high-risk groups. All hypothetical tests were two-sided, and *P* values<0.05 were considered significant.

## Supplementary Material

Supplementary Tables

## References

[1] Siegel RL, Miller KD, Jemal A. Cancer statistics, 2020. CA Cancer J Clin. 2020; 70:7–30. 10.3322/caac.21590 31912902

[2] Shield KD, Ferlay J, Jemal A, Sankaranarayanan R, Chaturvedi AK, Bray F, Soerjomataram I. The global incidence of lip, oral cavity, and pharyngeal cancers by subsite in 2012. CA Cancer J Clin. 2017; 67:51–64. 10.3322/caac.21384 28076666

[3] Ren ZH, Xu JL, Li B, Fan TF, Ji T, Zhang CP. Elective versus therapeutic neck dissection in node-negative oral cancer: evidence from five randomized controlled trials. Oral Oncol. 2015; 51:976–81. 10.1016/j.oraloncology.2015.08.009 26321080

[4] Birney E, Stamatoyannopoulos JA, Dutta A, Guigó R, Gingeras TR, Margulies EH, Weng Z, Snyder M, Dermitzakis ET, Thurman RE, Kuehn MS, Taylor CM, Neph S, et al, and ENCODE Project Consortium, and NISC Comparative Sequencing Program, and Baylor College of Medicine Human Genome Sequencing Center, and Washington University Genome Sequencing Center, and Broad Institute, and Children’s Hospital Oakland Research Institute. Identification and analysis of functional elements in 1% of the human genome by the ENCODE pilot project. Nature. 2007; 447:799–816. 10.1038/nature05874 17571346 PMC2212820

[5] Mercer TR, Dinger ME, Mattick JS. Long non-coding RNAs: insights into functions. Nat Rev Genet. 2009; 10:155–59. 10.1038/nrg2521 19188922

[6] Wu T, Zhang SY, Dong WJ, Wang M, Sun YB. The potential influence of long non-coding RNA PRKG1-AS1 on oral squamous cell carcinoma: a comprehensive study based on bioinformatics and *in vitro* validation. J Oral Pathol Med. 2020; 49:409–16. 10.1111/jop.12980 31788859

[7] Hu WY, Wei HY, Li KM, Wang RB, Xu XQ, Feng R. LINC00511 as a ceRNA promotes cell Malignant behaviors and correlates with prognosis of hepatocellular carcinoma patients by modulating miR-195/EYA1 axis. Biomed Pharmacother. 2020; 121:109642. 10.1016/j.biopha.2019.109642 31731191

[8] Quan D, Chen K, Zhang J, Guan Y, Yang D, Wu H, Wu S, Lv L. Identification of lncRNA NEAT1/miR-21/RRM2 axis as a novel biomarker in breast cancer. J Cell Physiol. 2020; 235:3372–81. 10.1002/jcp.29225 31621912 PMC13482681

[9] Cao W, Liu JN, Liu Z, Wang X, Han ZG, Ji T, Chen WT, Zou X. A three-lncRNA signature derived from the atlas of ncRNA in cancer (TANRIC) database predicts the survival of patients with head and neck squamous cell carcinoma. Oral Oncol. 2017; 65:94–101. 10.1016/j.oraloncology.2016.12.017 28109476

[10] Kopp F, Mendell JT. Functional classification and experimental dissection of long noncoding RNAs. Cell. 2018; 172:393–407. 10.1016/j.cell.2018.01.011 29373828 PMC5978744

[11] Rong L, Zhao R, Lu J. Highly expressed long non-coding RNA FOXD2-AS1 promotes non-small cell lung cancer progression via Wnt/β-catenin signaling. Biochem Biophys Res Commun. 2017; 484:586–91. 10.1016/j.bbrc.2017.01.141 28132805

[12] Zhu Y, Qiao L, Zhou Y, Ma N, Wang C, Zhou J. Long non-coding RNA FOXD2-AS1 contributes to colorectal cancer proliferation through its interaction with microRNA-185-5p. Cancer Sci. 2018; 109:2235–42. 10.1111/cas.13632 29737580 PMC6029818

[13] Xu TP, Wang WY, Ma P, Shuai Y, Zhao K, Wang YF, Li W, Xia R, Chen WM, Zhang EB, Shu YQ. Upregulation of the long noncoding RNA FOXD2-AS1 promotes carcinogenesis by epigenetically silencing EphB3 through EZH2 and LSD1, and predicts poor prognosis in gastric cancer. Oncogene. 2018; 37:5020–36. 10.1038/s41388-018-0308-y 29789713

[14] Yang X, Duan B, Zhou X. Long non-coding RNA FOXD2-AS1 functions as a tumor promoter in colorectal cancer by regulating EMT and notch signaling pathway. Eur Rev Med Pharmacol Sci. 2017; 21:3586–91. 28925486

[15] Ge P, Cao L, Yao YJ, Jing RJ, Wang W, Li HJ. lncRNA FOXD2-AS1 confers cisplatin resistance of non-small-cell lung cancer via regulation of miR185-5p-SIX1 axis. Onco Targets Ther. 2019; 12:6105–17. 10.2147/OTT.S197454 31534348 PMC6681567

[16] Ni W, Xia Y, Bi Y, Wen F, Hu D, Luo L. FoxD2-AS1 promotes glioma progression by regulating miR-185-5P/HMGA2 axis and PI3K/AKT signaling pathway. Aging (Albany NY). 2019; 11:1427–39. 10.18632/aging.101843 30860979 PMC6428107

[17] Liu X, Fu Q, Li S, Liang N, Li F, Li C, Sui C, Dionigi G, Sun H. LncRNA FOXD2-AS1 functions as a competing endogenous RNA to regulate TERT expression by sponging miR-7-5p in thyroid cancer. Front Endocrinol (Lausanne). 2019; 10:207. 10.3389/fendo.2019.00207 31024447 PMC6463795

[18] Xu Y, Wang J, Cai S, Chen G, Xiao N, Fu Y, Chen Q, Qiu S. PNCK depletion inhibits proliferation and induces apoptosis of human nasopharyngeal carcinoma cells *in vitro* and *in vivo*. J Cancer. 2019; 10:6925–32. 10.7150/jca.33698 31839828 PMC6909947

[19] Sun Q, Zhang SY, Zhao JF, Han XG, Wang HB, Sun ML. HIF-1α or HOTTIP/CTCF promotes head and neck squamous cell carcinoma progression and drug resistance by targeting HOXA9. Mol Ther Nucleic Acids. 2020; 20:164–75. 10.1016/j.omtn.2019.12.045 32169804 PMC7068198

[20] Ding WJ, Zhou M, Chen MM, Qu CY. HOXB8 promotes tumor metastasis and the epithelial-mesenchymal transition via ZEB2 targets in gastric cancer. J Cancer Res Clin Oncol. 2017; 143:385–97. 10.1007/s00432-016-2283-4 27761656 PMC11819265

[21] Li X, Lin H, Jiang F, Lou Y, Ji L, Li S. Knock-down of HOXB8 prohibits proliferation and migration of colorectal cancer cells via Wnt/β-catenin signaling pathway. Med Sci Monit. 2019; 25:711–20. 10.12659/MSM.912218 30677006 PMC6357822

[22] López-Sánchez LM, Jurado-Gámez B, Feu-Collado N, Valverde A, Cañas A, Fernández-Rueda JL, Aranda E, Rodríguez-Ariza A. Exhaled breath condensate biomarkers for the early diagnosis of lung cancer using proteomics. Am J Physiol Lung Cell Mol Physiol. 2017; 313:L664–76. 10.1152/ajplung.00119.2017 28619761

[23] Hu HB, Yang XP, Zhou PX, Yang XA, Yin B. High expression of keratin 6C is associated with poor prognosis and accelerates cancer proliferation and migration by modulating epithelial-mesenchymal transition in lung adenocarcinoma. Genes Genomics. 2020; 42:179–88. 10.1007/s13258-019-00889-5 31768767

[24] Freed DM, Bessman NJ, Kiyatkin A, Salazar-Cavazos E, Byrne PO, Moore JO, Valley CC, Ferguson KM, Leahy DJ, Lidke DS, Lemmon MA. EGFR ligands differentially stabilize receptor dimers to specify signaling kinetics. Cell. 2017; 171:683–95.e18. 10.1016/j.cell.2017.09.017 28988771 PMC5650921

[25] Abi Zamer B, Mahfood M, Saleh B, Al Mutery AF, Tlili A. Novel mutation in the DSG1 gene causes autosomal-dominant striate palmoplantar keratoderma in a large Syrian family. Ann Hum Genet. 2019; 83:472–76. 10.1111/ahg.12335 31192455

[26] Nomura T, Takeda M, Peh JT, Miyauchi T, Suzuki S, Fujita Y, Uesugi T, Shimizu H. Loss-of-function mutation in DSG1 underlies focal palmoplantar keratoderma. J Eur Acad Dermatol Venereol. 2019; 33:e137–38. 10.1111/jdv.15349 30451323

[27] Wong MP, Cheang M, Yorida E, Coldman A, Gilks CB, Huntsman D, Berean K. Loss of desmoglein 1 expression associated with worse prognosis in head and neck squamous cell carcinoma patients. Pathology. 2008; 40:611–16. 10.1080/00313020802320614 18752129

[28] Valenzuela-Iglesias A, Burks HE, Arnette CR, Yalamanchili A, Nekrasova O, Godsel LM, Green KJ. Desmoglein 1 regulates invadopodia by suppressing EGFR/Erk signaling in an erbin-dependent manner. Mol Cancer Res. 2019; 17:1195–206. 10.1158/1541-7786.MCR-18-0048 30655320 PMC6581214

[29] Guo X, Gudas LJ. Metabolism of all-trans-retinol in normal human cell strains and squamous cell carcinoma (SCC) lines from the oral cavity and skin: reduced esterification of retinol in SCC lines. Cancer Res. 1998; 58:166–76. 9426073

[30] Cui J, Gong M, He Y, Li Q, He T, Bi Y. All-trans retinoic acid inhibits proliferation, migration, invasion and induces differentiation of hepa1-6 cells through reversing EMT *in vitro*. Int J Oncol. 2016; 48:349–57. 10.3892/ijo.2015.3235 26548461

[31] Shi G, Zheng X, Wu X, Wang S, Wang Y, Xing F. All-trans retinoic acid reverses epithelial-mesenchymal transition in paclitaxel-resistant cells by inhibiting nuclear factor kappa B and upregulating gap junctions. Cancer Sci. 2019; 110:379–88. 10.1111/cas.13855 30375704 PMC6317959

[32] Kent LN, Leone G. The broken cycle: E2F dysfunction in cancer. Nat Rev Cancer. 2019; 19:326–38. 10.1038/s41568-019-0143-7 31053804

[33] Nevins JR. The Rb/E2F pathway and cancer. Hum Mol Genet. 2001; 10:699–703. 10.1093/hmg/10.7.699 11257102

[34] Fu J, Lv H, Guan H, Ma X, Ji M, He N, Shi B, Hou P. Metallothionein 1G functions as a tumor suppressor in thyroid cancer through modulating the PI3K/Akt signaling pathway. BMC Cancer. 2013; 13:462. 10.1186/1471-2407-13-462 24098937 PMC3851544

[35] Kent LN, Rakijas JB, Pandit SK, Westendorp B, Chen HZ, Huntington JT, Tang X, Bae S, Srivastava A, Senapati S, Koivisto C, Martin CK, Cuitino MC, et al. E2f8 mediates tumor suppression in postnatal liver development. J Clin Invest. 2016; 126:2955–69. 10.1172/JCI85506 27454291 PMC4966321

[36] Kent LN, Bae S, Tsai SY, Tang X, Srivastava A, Koivisto C, Martin CK, Ridolfi E, Miller GC, Zorko SM, Plevris E, Hadjiyannis Y, Perez M, et al. Dosage-dependent copy number gains in E2f1 and E2f3 drive hepatocellular carcinoma. J Clin Invest. 2017; 127:830–42. 10.1172/JCI87583 28134624 PMC5330731

[37] Su F, He W, Chen C, Liu M, Liu H, Xue F, Bi J, Xu D, Zhao Y, Huang J, Lin T, Jiang C. The long non-coding RNA FOXD2-AS1 promotes bladder cancer progression and recurrence through a positive feedback loop with Akt and E2F1. Cell Death Dis. 2018; 9:233. 10.1038/s41419-018-0275-9 29445134 PMC5833400

[38] Chirica M, Le Bourhis L, Lehmann-Che J, Chardiny V, Bouhidel F, Foulboeuf L, Gornet JM, Lourenco N, Dulphy N, Toubert A, Allez M. Phenotypic analysis of T cells infiltrating colon cancers: correlations with oncogenetic status. Oncoimmunology. 2015; 4:e1016698. 10.1080/2162402X.2015.1016698 26405567 PMC4570110

[39] Lee AM, Clear AJ, Calaminici M, Davies AJ, Jordan S, MacDougall F, Matthews J, Norton AJ, Gribben JG, Lister TA, Goff LK. Number of CD4+ cells and location of forkhead box protein P3-positive cells in diagnostic follicular lymphoma tissue microarrays correlates with outcome. J Clin Oncol. 2006; 24:5052–59. 10.1200/JCO.2006.06.4642 17033038

[40] Englund E, Reitsma B, King BC, Escudero-Esparza A, Owen S, Orimo A, Okroj M, Anagnostaki L, Jiang WG, Jirström K, Blom AM. The human complement inhibitor sushi domain-containing protein 4 (SUSD4) expression in tumor cells and infiltrating T cells is associated with better prognosis of breast cancer patients. BMC Cancer. 2015; 15:737. 10.1186/s12885-015-1734-7 26480818 PMC4615997

[41] Love MI, Huber W, Anders S. Moderated estimation of fold change and dispersion for RNA-seq data with DESeq2. Genome Biol. 2014; 15:550. 10.1186/s13059-014-0550-8 25516281 PMC4302049

[42] Zhou Y, Zhou B, Pache L, Chang M, Khodabakhshi AH, Tanaseichuk O, Benner C, Chanda SK. Metascape provides a biologist-oriented resource for the analysis of systems-level datasets. Nat Commun. 2019; 10:1523. 10.1038/s41467-019-09234-6 30944313 PMC6447622

[43] Yu G, Wang LG, Han Y, He QY. clusterProfiler: an R package for comparing biological themes among gene clusters. OMICS. 2012; 16:284–87. 10.1089/omi.2011.0118 22455463 PMC3339379

[44] Bindea G, Mlecnik B, Tosolini M, Kirilovsky A, Waldner M, Obenauf AC, Angell H, Fredriksen T, Lafontaine L, Berger A, Bruneval P, Fridman WH, Becker C, et al. Spatiotemporal dynamics of intratumoral immune cells reveal the immune landscape in human cancer. Immunity. 2013; 39:782–95. 10.1016/j.immuni.2013.10.003 24138885

