# Supplementary material for: Dysregulation of FOXD2-AS1 promotes cell proliferation and migration and predicts poor prognosis in oral squamous cell carcinoma: a study based on TCGA data
Source: Aging (Albany NY). 2020 Dec 9;13(2):2379–96. doi: 10.18632/aging.202268 (PMC7880351; doi:10.18632/aging.202268)
Supplement: Supplementary Tables [file aging-13-202268-s001.pdf]

SUPPLEMENTARY TABLES

Supplementary Table 1. Sequences of the qRT-PCR primers.

Gene	Forward primer	Reverse primer
FOXD2-AS1	GTGGGGAATGAGGATGGGTG	TGCCGCTGGAGTATTCTTGG
βactin	CCTGGCACCCAGCACAAT	GGGCCGGACTCGTCATAC
MALAT1	CCTAACCAGGCATAACACAGAAT	CGAATGGCTTTGTCTCCGAA
PNCK	CGCCCTTTGAGGACTCGAAG	GGCCACATATCCAGGGGTC
HOXB9	CCATTTCTGGGACGCTTAGCA	TGTAAGGGTGGTAGACGGACG
HOXB8	GACCCCGGCAATTTCTACGG	ATCCAGGGGAAGAGCTGTGT
KRT6C	ACTTCCTGAGAGCCTTGTATGA	ATCTCCTCGTATTGGGCCTTG
EPGN	ATGGCTTTGGGAGTTCCAATATC	TCCTTCTATGTTGTCAGCTTGC
DSG1	AACCCAATCGCCAAAATTCACT	ACCTCTCGATCAACTATGGATGT
E-cadherin	CCTTCCTCCCAATACATCTC	TCACACACGCTGACCTCTAA
N-cadherin	AGGCCCCAGGGAATCTTTCA	GCCTCTCCCTGAATAACTGGG
Snail1	CCCCAATCGGAAGCCTAACT	CGTAGGGCTGCTGGAAGGTA

Supplementary Table 2. Sequences of the siRNA and smart silence kit.

Product	Sequences
SiRNA	GCGAAGAGTACGTTGCTAT
	CAGCCAAGAATACTCCAGCG
	TAAGCCTCATAGAAGCAAAG
Smart Silence Kit	ATCCACTCCACCCACGCCTT
	GCTTCCAGGTATGTGGGAA
	GCGACCTGTGTGTACCATA
	CCACTCTTCGCTTATGTTT
